# High Dietary Phosphorus Impairs Bone Microarchitecture and Induces Alterations in the LGR4–R-Spondins Axis in Rats with Normal Renal Function

**DOI:** 10.3390/nu17122049

**Published:** 2025-06-19

**Authors:** Sara Fernández-Villabrille, Francisco Baena-Huerta, Laura Suárez-Fernández, Elena Nefyodova, Paula Calvó, Nerea González-García, Helena Gil-Peña, Carlos Gómez-Alonso, Cristina Alonso-Montes, Manuel Naves-Díaz, Christa Maes, Natalia Carrillo-López, Sara Panizo

**Affiliations:** 1 Metabolismo Óseo, Vascular y Enfermedades Inflamatorias Crónicas, Instituto de Investigación Sanitaria del Principado de Asturias (ISPA), 33011 Oviedo, Spain; 2 Redes de Investigación Cooperativa Orientadas a Resultados en Salud, RICORS2040-Renal, 33011 Oviedo, Spain; 3 Unidad de Metabolismo Óseo, Unidad de Gestión Clínica de Medicina Interna, Hospital Universitario Central de Asturias, 33011 Oviedo, Spain; 4 Laboratory of Skeletal Cell Biology and Physiology (SCEBP), Skeletal Biology and Engineering Research Center (SBE), Department of Development and Regeneration, KU Leuven, 3000 Leuven, Belgium; 5 Area de Gestión Clínica de la Infancia y Adolescencia, Hospital Universitario Central de Asturias (HUCA), Instituto de Investigación Sanitaria del Principado de Asturias (ISPA), 33011 Oviedo, Spain; 6 Programa de Doctorado en Ciencias de la Salud, Universidad de Oviedo, 33006 Oviedo, Spain

**Keywords:** phosphorus, parathyroid hormone, Receptor Activator of Nuclear factor Kappa-B ligand, osteoprotegerin, leucine rich repeat containing G protein-coupled receptor 4, R-spondins, trabecular bone

## Abstract

**Background:** The increasing prevalence of processed foods has significantly elevated dietary phosphorus intake globally, posing a risk to skeletal health. Elevated serum phosphate promotes parathyroid hormone (PTH) release, leading to bone resorption and decreased bone formation. **Objective**: This study investigated the influence of chronically elevated phosphorus intake on bone structure in rats with normal renal function, focusing on the Receptor Activator of Nuclear factor Kappa-B (RANK)/RANK ligand (RANKL)/osteoprotegerin (OPG) pathway and its related components, leucine rich repeat containing G protein-coupled receptor 4 (LGR4), and R-spondins (RSPOs). **Methods:** Rats were fed a high-phosphorus diet, followed by assessment of the bone microstructure and of the expression of key signalling molecules. **Results:** Elevated phosphorus intake induced significant bone deterioration, particularly in the trabecular bone compartment, associated with alterations in the RANK/RANKL/OPG pathway and in the LGR4 and RSPO1 and RSPO4 signalling components in bone. Moreover, we also observed changes in RANKL, RSPO1 and RSPO4 serum levels in the rats that had received a high-phosphorus diet. **Conclusions:** These findings highlight the detrimental impact of excessive dietary phosphorus on skeletal health, even without renal impairment, and suggest that components of this pathway, particularly RSPO1 and RSPO4, could serve as potential biomarkers of bone deterioration. The widespread consumption of phosphorus-rich processed foods underscores the importance of nutritional education to mitigate these skeletal risks in industrialized populations.

## 1. Introduction

The increasing consumption of processed foods and the extensive use of food additives have led to a substantial rise in dietary phosphorus intake across global populations. Inorganic phosphorus, a key component of many food additives, demonstrates significantly higher intestinal absorption (approximately 90–100%) compared to phosphorus from animal sources (40–60%) and plant sources (10–30%). Excessive phosphorus intake has been linked to detrimental effects on various physiological systems and represents a significant risk to skeletal health [1]. This heightened phosphorus consumption disrupts the delicate equilibrium of mineral homeostasis, a critical factor in bone integrity. Specifically, elevated serum phosphate levels directly stimulate parathyroid hormone (PTH) secretion, a key regulator of the calcium and phosphate balance [2,3,4,5]. This stimulation initiates a cascade of harmful effects, including enhanced bone resorption and decreased bone formation, compromising bone mineral density (BMD) [6]. Furthermore, hyperphosphatemia not only directly impacts PTH secretion but also suppresses the renal production of 1,25-dihydroxyvitamin D (calcitriol), a crucial hormone for intestinal calcium absorption, thereby exacerbating bone mineral loss and further disrupting mineral homeostasis [7]. Moreover, phosphate accumulation has been shown to exert direct effects on bone cells, impairing osteoblast function and simultaneously promoting osteoclast differentiation through the action on the Receptor Activator of Nuclear factor Kappa-B (RANK)/RANK ligand (RANKL)/osteoprotegerin (OPG) signalling pathway [8,9].

The RANK/RANKL/OPG system plays a central role in the dynamic process of bone remodelling. RANKL, produced primarily by osteoblasts, serves as a key signalling molecule that stimulates osteoclastogenesis via its interaction with the RANK receptor, leading to bone resorption. Conversely, OPG functions as a soluble receptor, effectively inhibiting RANKL binding to RANK and thus suppressing osteoclast activity [10]. Beyond this established system, leucine-rich repeat-containing G protein-coupled receptor 4 (LGR4) has been identified as a significant modulator of bone metabolism being expressed both in osteoblasts and osteoclasts. LGR4 modulates osteoclast differentiation and activation by competitively binding to RANKL, thereby interrupting the canonical RANKL-RANK-Tumour Necrosis Factor Receptor Associated Factor 6 (TRAF6) signalling pathway. This disruption reduces NFATC1 activity, consequently diminishing RANKL-driven bone resorption. In osteoblasts, LGR4 additionally activates the cAMP–PKA–CREB signalling pathway. This activation controls the expression of the transcription factor ATF4 and various osteogenic proteins, including osteocalcin, bone sialoprotein, and collagen type [11,12]. Notably, LGR4 can also promote osteoblast maturation by functioning as a receptor for R-spondins (RSPOs), a family of secreted proteins that potentiate Wnt signalling, which is a critical pathway for osteoblast differentiation and bone formation. Thus, LGR4 signalling overall positively influences bone homeostasis [13,14].

Given the intricate interplay of these molecular pathways in skeletal physiology and the role of elevated phosphorus in disrupting their function, the present study was designed to examine the association among high phosphorus intake, bone structure, and LGR4. Specifically, we evaluated the impact of elevated phosphorus on bone remodelling markers, the RANKL/OPG/LGR4 pathway and the LGR4 ligands, RSPOs, to explore their potential contributions to the mechanisms by which dietary phosphorus influences bone health.

## 2. Materials and Methods

### 2.1. Design

This study employed a combined experimental approach using both in vivo and in vitro models to investigate the effects of phosphate and PTH on bone metabolism, with a specific focus on the RANKL/OPG/LGR4/RSPOs signalling pathway. The in vivo component involved adult rats with normal renal function, which were randomly assigned to two dietary regimens differing in phosphorus content. This model was used to evaluate the impact of dietary phosphorus on bone microarchitecture, serum, and urinary biochemical markers, and the expression of genes related to bone remodelling. In parallel, the in vitro model utilised the UMR-106 osteoblast-like cell line, which was exposed to phosphate and/or PTH to examine direct cellular effects on the same signalling pathway. All procedures involving animals were approved by the Laboratory Animal Ethics Committee at the University of Oviedo (PROAE 14/2021) and conducted in accordance with the National Institutes of Health’s Guide for the Care and Use of Laboratory Animals.

### 2.2. Phosphate and PTH Treatment of Osteoblast Cultures

Rat osteoblastic cell line UMR-106 (ATCC, Manassas, VA, USA) was exposed to an elevated phosphate concentration (3 mM) and PTH 1-34 (10^−8^ M, Sigma-Aldrich, St. Louis, MO, USA), both individually and in combination, for 48 h. Inorganic phosphate and PTH were prepared in distilled water and PBS, respectively, and added to Dulbecco Modified Eagle Medium (DMEM) supplemented with 10% foetal bovine serum (FBS) and 1% penicillin/streptomycin (Lonza, Alsace, France). Control cells were cultured in DMEM medium containing 1.8 mM calcium and 1 mM phosphate (control group). The phosphate condition consisted of 1.8 mM calcium and 3 mM phosphate (phosphate group); the PTH condition included 1.8 mM calcium, 1 mM phosphate and PTH 10^−8^ M (PTH group); and the combination phosphate + PTH condition included 1.8 mM calcium, 3 mM phosphate and PTH 10^−8^ M (phosphate + PTH group). Following treatment, protein expression levels of RANKL, OPG, LGR4, and RSPOs 1–4 were analysed via Western blot. All experiments were conducted in triplicate.

### 2.3. Western Blot

For protein analysis, total protein extracts were obtained from UMR-106 cells and quantified utilizing the DC protein assay (Bio-Rad, Hercules, CA, USA). Samples containing 30 µg of protein underwent separation via 10% SDS-polypolyacrylamide gel electrophoresis. Proteins were then electroblotted onto polyvinylidene difluoride membranes (Hybond; Amersham, UK). Western blotting was performed according to the manufacturers’ guidelines using the indicated specific antibodies: RANKL ((G1) sc-377079; 1:500; Santa Cruz Biotechnology, Dallas, TX, USA), OPG ((E-10) sc-390518; 1:500; Santa Cruz Biotechnology), LGR4 ((C-12) sc-390630; 1:500; Santa Cruz Biotechnology), RSPO1 (PA5-20749; 1:1000; Thermo Fisher, Walthman, MA, USA), RSPO2 ((aa22-243) LS-C754556; 1:500; LifeSpan BioSciences, Lynnwood, WA, USA), RSPO3 (PA-100324; 1:500; Thermo Fisher), RSPO4 (ab189515; 1:1000; Abcam, Cambridge, UK) and GAPDH ((FL-335) sc-25778; 1:3000; Santa Cruz Biotechnology). Secondary antibody binding was detected using the ECL Western Blotting Detection Kit (Amersham, UK). Chemiluminescent signals were visualized and quantified with the ChemiDoc Gel Imaging System Model XRS (Bio-Rad) using Quantity One 1-D analysis software version 4 (Bio-Rad).

### 2.4. Animal Model

Four-month-old male Wistar rats, weighing between 350 and 400 g, were allocated into two distinct dietary groups: one group received a standard rodent diet containing normal phosphorus levels (NP; 0.6% phosphorus, 0.6% calcium, and 20% protein. Envigo, Indianapolis, IN, USA) (*n* = 8) and the second group received a high phosphorus diet (HP; 0.9% phosphorus, 0.6% calcium, and 20% protein. Envigo) (*n* = 10). The animals were housed in wire cages with *ad libitum* access to food and water. After four months, the animals were transferred to metabolic cages for a 24-h urine collection before being euthanized by exsanguination under isoflurane anaesthesia. Serum samples were then collected for later analyses. Both tibias were collected; the left tibia was utilised for microarchitectural analysis of the trabecular and cortical bone, and the right tibia for the assessment of mRNA expression changes.

### 2.5. Biochemical Markers

Serum and urine levels of creatinine, phosphate and calcium, and serum calcitriol were quantified using a multichannel autoanalyzer (Hitachi 717, Boehringer, Mannheim, Germany). Serum PTH and fibroblast growth factor-23 (FGF23) concentrations were determined using sandwich enzyme-linked immunosorbent assay (ELISA) kits (Rat Intact PTH ELISA Kit (REF:60-2500); Quidel, San Diego, CA, USA and Mouse/Rat Intact FGF23 Kit (REF:60-6800); Immutopics, San Clemente, CA, USA), in accordance with the manufacturers’ instructions. The fractional excretion of phosphate or calcium was calculated using the formula: [(urine concentration of the molecule * serum creatinine)/(serum concentration of the molecule * urine creatinine)].

Serum levels of RANKL, OPG, LGR4, RSPO1 and RSPO4 were measured by ELISA kits (RANKL (REF:MTR00) and OPG (REF:MOP00): R&D Systems, Minneapolis, MN, USA; LGR4 (REF:MBS9342881), RSPO1 (REF:MBS053652) and RSPO4 (REF:MBS109763): MyBioSource, San Diego, CA, USA).

### 2.6. Micro-Computed Tomography (Micro-CT)

The proximal metaphysis to mid-diaphysis region of the left tibia was scanned using micro-computed tomography (micro-CT; SkyScan 1174, Aartselaar, Belgium) to assess trabecular and cortical bone microarchitecture. Subsequently, morphometric 2D and 3D analyses were conducted utilizing the manufacturer-provided software (CTAn 1.19, Bruker, Billerica, MA, USA). The main parameters evaluated were as follows: Bone Mineral Density (BMD), which quantifies the amount of mineral—primarily calcium—present in each area of bone, and is commonly used to assess trabecular bone by averaging the bone and marrow content within the medullary volume of interest (a three-dimensional region of the bone that has been selected for analysis); Tissue Mineral Density (TMD) measures the mineral density within the bone tissue itself, excluding the marrow space, providing a more accurate assessment of cortical bone mineral density; Trabecular Number (Tb.N), representing the number of trabeculae per unit volume; Trabecular Thickness (Tb.Th), indicating the average thickness of trabeculae; Trabecular Separation (Tb.Sp), measuring the average distance between trabeculae; Connectivity Density (Conn.Dn), indicating the number of trabecular connections per unit volume; Trabecular Bone Surface Density (BS/TV), representing the ratio of trabecular bone surface to total volume; Trabecular Bone Volume/Total Volume (BV/TV), the ratio of bone volume to total volume within the trabecular region; Structure Model Index (SMI), describing trabecular shape; Trabecular Pattern Factor (Tb.Pf), measuring trabecular network complexity; Trabecular Porosity (Tb.Po), which measures the percentage of pore volume within the trabecular bone; Cortical Thickness (Ct.Th), representing the average thickness of the cortical bone; Cortical Separation (Ct.Po. Dm), indicating the average distance between cortical pores; Cortical Bone Porosity (Ct.Po), which measures the percentage of pore volume within the cortical bone; Cortical Bone Volume (BV/TV), the ratio of bone volume to total volume within the cortical region; and Bone Surface Density (BS/BV), which refers to the ratio of the bone surface area to the bone volume.

### 2.7. Bone Histology and Histomorphometry

The one-third proximal segment of the left tibia, initially fixed in 10% formaldehyde and subsequently in 70% ethanol, was dehydrated and embedded in methyl methacrylate according to a low-temperature embedding procedure that preserves protein antigenicity in the tissue, as described by Erben [15]. Longitudinal sections of 5 μm were obtained using a Microm HM355 S microtome (Walldorf, Germany). After deplastification, Von Kossa, toluidine blue, and Tartrate-Resistant Acid Phosphatase (TRAP) staining were performed using standard protocols [16] as described before [17,18]. The osteoblast surface was measured in samples stained with a 2% solution of toluidine blue (Sigma-Aldrich, St. Louis, MO, USA) in the buffer 8.2 mM citric acid, 5.3 mM disodium phosphate, distilled water, pH = 3.7. The osteoclast number and trabecular surface cover by osteoclasts were determined by TRAP staining using 0.5 mg/mL naphthol AS-MX phosphate (Sigma-Aldrich, St. Louis, MO, USA) and 1.1 mg/mL fast red violet (Sigma-Aldrich) followed by counterstaining with fast green. Two non-consecutive sections per sample were examined using an Olympus IX83 inverted microscope equipped with a DP73 camera (Olympus, Shinjuku, Tokyo, Japan). Images were analysed using OsteoMeasure software 4.3.0.1 (OsteoMetrics, Decatur, GA, USA) according to the standardised guidelines [19] as before [17,18].

### 2.8. RNA Isolation, Reverse Transcription and Quantitative PCR

The proximal metaphysis to mid-diaphysis region of the right tibia from the rats was homogenised in liquid nitrogen using a mortar (OmniHT [Omni Internacional, Kennesaw, GA, USA]) in TRI Reagent (Sigma-Aldrich, St. Louis, MO, USA) adhering to the manufacturer’s instructions. RNA concentration and purity were assessed via UV-Vis Spectrophotometry (NanoDrop Technologies, Wilmington, DE, USA) at wavelengths of 260 and 280 nm. Reverse transcription was performed with a High-Capacity cDNA Reverse Transcription Kit (Applied Biosystems, Waltham, MA, USA). Real-time quantitative PCR (RT-qPCR) was performed on the resultant cDNA using TaqMan Assays (Applied Biosystems) on a Quant Studio 3 (Applied Biosystems) to quantify the expression levels of *Rank* (Rn04340164_m1), *Rankl* (Rn00589289_m1), *Opg* (Rn00563499_m1), *Lgr4* (Rn00597924_m1), *Rspo1* (Rn01517010_m1), *Rspo2* (Rn01505834_m1), *Rspo3* (Rn01537475_m1), and *Rspo4* (Rn06186317_m1) (Thermo Fisher Scientific, Waltham, MA, USA), with glyceraldehyde-3-phosphate dehydrogenase (*Gapdh*, Rn99999916_s1, Thermo Fisher Scientific) serving as the housekeeping gene. The relative quantification of genes was performed by comparing threshold cycles using the ΔΔ cycle threshold method [20].

### 2.9. Immunohistochemistry

The localisations of RANK, RANKL, OPG, LGR4, RSPO1, and RSPO4 in bone were assessed by immunohistochemistry in 4-µm deplastified sections from the left tibia embedded in methyl methacrylate. Samples were incubated thirty minutes at room temperature with primary antibodies against RANK (ab305233; 1:200 dilution; Abcam), RANKL (ab62516; 1:200; Abcam), OPG (PA5-86053; 1:1000; Invitrogen, San Diego, CA, USA), LGR4 (PA5-109908; 1:200; Invitrogen), RSPO1 (PA5-20749; 1:600; Invitrogen), and RSPO4 (ab189515; 1:10000; Abcam), and then washed and incubated with the secondary antibody following the manufacturer’s instructions (EnVision Flex Dako; Agilent, Santa Clara, CA, USA). The resulting stained area was quantified using an optical microscope (model DMRXA2; Leica Microsystems, Wetzlar, Germany) equipped with a digital video camera (model Dc-100; Leica Microsystems) and an image analysis system running ImageJ 1.53k software. Two operators, blinded to the experimental groups, performed all measurements. Results are presented as the percentage of the stained area relative to the total area. Negative control, processed without the primary antibody, was included to establish the minimum level of staining intensity.

### 2.10. Statistical Analysis

Data are presented as median and interquartile range (IQR). Groups were compared using the non-parametric Mann–Whitney U test. For comparisons involving more than two groups, the Kruskal–Wallis test was employed, followed by post hoc analyses Dunn’s Multiple Comparison test. All analyses were conducted using R software version 4.1.1.

## 3. Results

### 3.1. Phosphate and PTH Modulate Bone Remodelling Markers of the RANKL/OPG/LGR4 Axis and RSPOs Family in UMR-106 Osteoblasts

**Figure 1:**
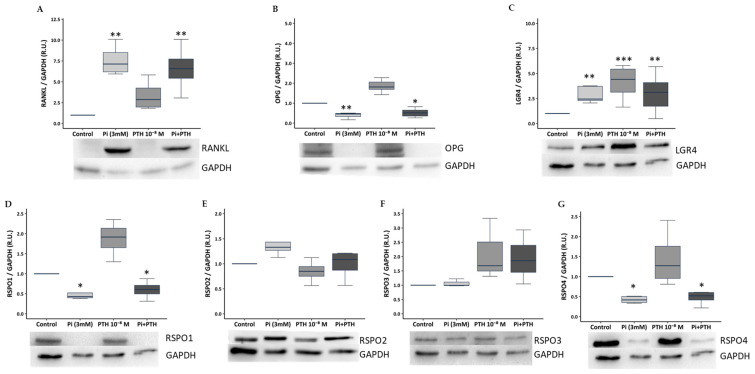
Effect of high phosphate (Pi, 3 mM phosphate) and high PTH (10⁻⁸ M) on rat osteoblast cell line UMR-106 after 48 h of exposure. Quantification of protein levels determined by Western blot and representative images of: (**A**) RANKL, (**B**) OPG, (**C**) LGR4, (**D**) RSPO1, (**E**) RSPO2, (**F**) RSPO3, and (**G**) RSPO4. Data are presented as median [interquartile range]. R.U.: relative units. * *p* < 0.05, ** *p* < 0.01, *** *p* < 0.001 versus control (1 mM phosphate). All groups are compared from the control group that is normalised to the unit.

The rat osteoblast cell line UMR-106 exposed to 3 mM phosphate for 48 h exhibited significant increases in RANKL and LGR4 protein levels (Figure 1A,C) and decreases in OPG, RSPO1, and RSPO4 (Figure 1B,D,G) versus control cells. Exposure of UMR-106 to PTH 10^−8^ M resulted in a significant increase in LGR4 expression (Figure 1C). Finally, exposure to the combination of 3 mM phosphate and 10^−8^ M PTH led to a significant increase in LGR4 and RANKL pathway components, along with a decrease in OPG, RSPO1, and RSPO4 compared to control. No significant changes in RSPO2 and RSPO3 were observed under any of the conditions employed (Figure 1E,F).

### 3.2. High Dietary Phosphorus Alters Mineral Homeostasis in Rats

Rats maintained on a high phosphorus (HP) diet exhibited significantly elevated serum phosphate, PTH and calcitriol concentrations compared to rats maintained on a normal phosphorus (NP) diet (Table 1). No statistically significant differences were observed in serum calcium and FGF23 levels, or creatinine clearance (Table 1). The fractional excretion of phosphorus was markedly increased in the HP diet group, whereas the fractional excretion of calcium was decreased compared to the NP diet group (Table 1).

### 3.3. Influence of High Dietary Phosphorus on Bone Mass and Trabecular and Cortical Bone Architecture

BMD exhibited a significant decrease in the rats fed a HP diet compared to rats maintained on a NP diet. However, TMD showed no significant differences between the groups (Table 2).

In the trabecular bone region, micro-CT analysis revealed that Tb.N, BS/TV, Conn.Dn, and BV/TV were significantly lower in the HP diet animal group. This group also exhibited higher Tb.Th, Tb.Sp, Tb.Po, and SMI (Table 2). Micro-CT analysis showed no significant differences in Tb.Pf (Table 2).

Regarding the cortical bone compartment measurement, Ct.Th, Ct.Po.Dm, Ct.Po, BV/TV, BS/BV showed no significant differences between rats fed a NP and HP diet (Table 2).

**Figure 2:**
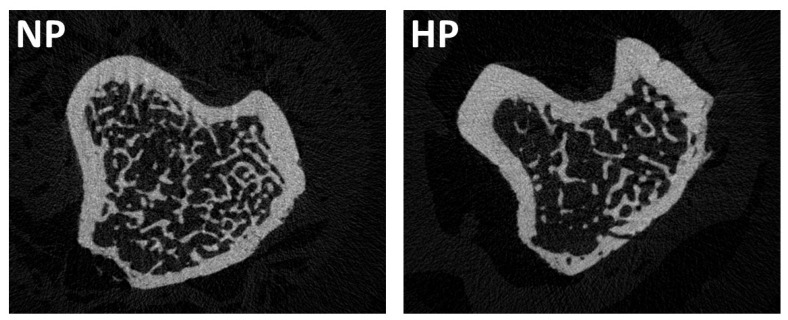
Representative images illustrating the effects of dietary phosphorus intake on tibia microarchitecture by micro-CT. NP: normal phosphorus diet (0.6% phosphorus), and HP: high phosphorus diet (0.9% phosphorus).

Figure 2 showed a representative image of the micro-CT in each group of rats.

These results indicate that high dietary phosphorus induces a significant reduction in bone mineral density and deteriorates trabecular microarchitecture, while cortical bone parameters remain unaffected.

### 3.4. Influence of High Dietary Phosphorus on Bone Morphology, Osteoblast and Osteoclast Number

**Figure 3:**
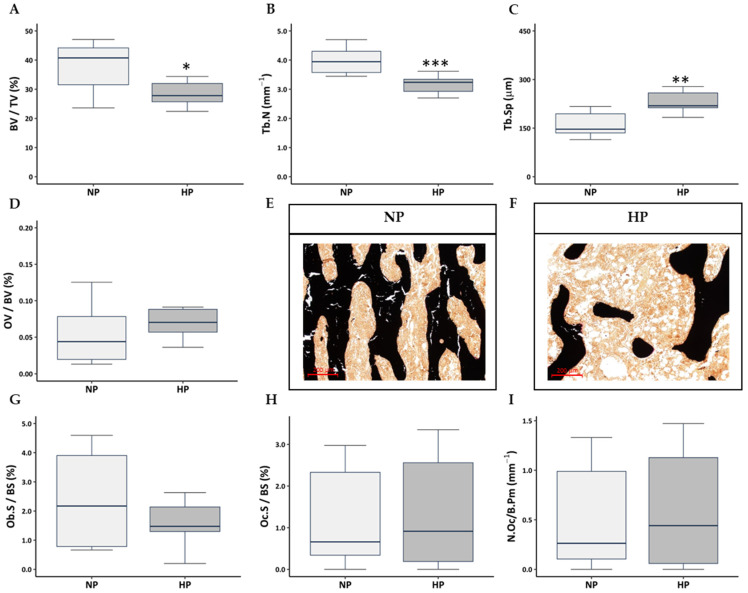
Effect of dietary phosphorus level on trabecular bone microarchitecture. (**A**) Trabecular bone volume (BV/TV), (**B**) trabecular number (Tb.N), (**C**) trabecular separation (Tb.Sp), (**D**) volume of non-mineralised bone (osteoid; OV/BV), as measured on methyl methacrylate sections stained for Von Kossa/Von Gieson, (**E**) representative image of the Von Kossa/Von Gieson staining of the tibia from NP and (**F**) HP rats, (**G**) osteoblast-covered bone surface (Ob.S/BS) analysed by toluidine blue staining, (**H**) osteoclast-covered bone surface (Oc.S/BS), and (**I**) number of osteoclasts per bone perimeter (N.Oc/B.Pm) analysed by tartrate-resistant acid phosphatase (TRAP) staining. NP: normal phosphorus diet (0.6% phosphorus), and HP: high phosphorus diet (0.9% phosphorus). Each inset indicates the relative scale in µm. Data are presented as median [interquartile range] * *p* < 0.05, ** *p* < 0.01, *** *p* < 0.001 versus NP.

As revealed by histomorphometric analysis of the tibias, rats fed a HP diet showed significant alterations in trabecular bone microarchitecture compared to rats fed on a NP diet, characterised by a reduction in BV/TV (Figure 3A,E,F) and Tb.N (Figure 3B,E,F), along with an increase in Tb.Sp (Figure 3C,E,F). No significant differences were detected in the OV/BV between the diet groups (Figure 3D).

Despite the change in bone mass, further histomorphometric analysis of osteoblast and osteoclast parameters revealed no statistically significant differences between NP and HP diet groups. Neither the osteoblast-covered bone surface (Ob.S/BS), nor the osteoclast-covered bone surface Oc.S/BS and number of osteoclasts per bone perimeter (N.Oc/B.Pm) (Figure 3G–I) were significantly different between the diet groups.

Thus, high dietary phosphorus impairs trabecular bone microarchitecture by reducing bone volume and trabecular number and increasing trabecular separation, yet without noticeably affecting the osteoid volume or static histomorphometric indices of osteoblast and osteoclast numbers.

### 3.5. Influence of High Dietary Phosphorus on the RANKL/OPG/LGR4 Axis and RSPO Expression in Rat Tibia

**Figure 4:**
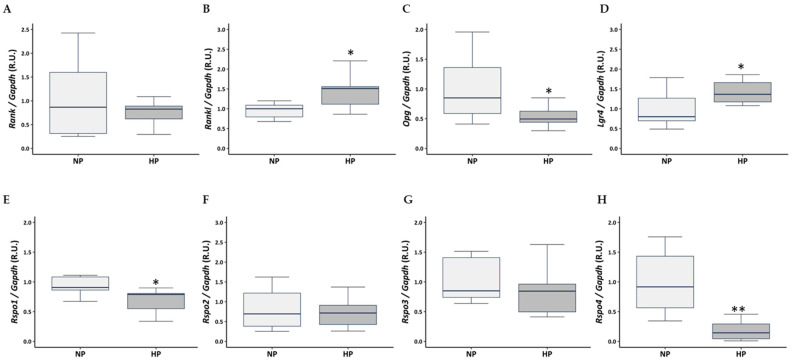
Effect of dietary phosphorus intake on gene expression of RANK, RANKL and OPG axis, and of the related components LGR4 and RSPOs in tibia. Gene expression was evaluated by qRT-PCR for (**A**) *Rank*, (**B**) *Rankl*, (**C**) *Opg*, (**D**) *Lgr4*, (**E**) *Rspo1*, (**F**) *Rspo2*, (**G**) *Rspo3*, (**H**) *Rspo4*. NP: normal phosphorus diet (0.6% phosphorus), and HP: high phosphorus diet (0.9% phosphorus). Data are presented as median [interquartile range]. R.U.: relative units. * *p* < 0.05, ** *p* < 0.01 versus NP.

In the tibia of rats fed a HP diet compared to rats fed a NP diet, no significant changes were observed in the mRNA expression levels of *Rank* (Figure 4A). However, a significant increase in *Rankl* (Figure 4B) and *Lgr4* (Figure 4D) mRNA expression was observed, while a significant decrease was observed in the expression of *Opg* (Figure 4C), *Rspo1* (Figure 4E), and *Rspo4* (Figure 4H). No significant changes were observed in *Rspo2* and *Rspo3* gene expression (Figure 4F,G).

**Figure 5:**
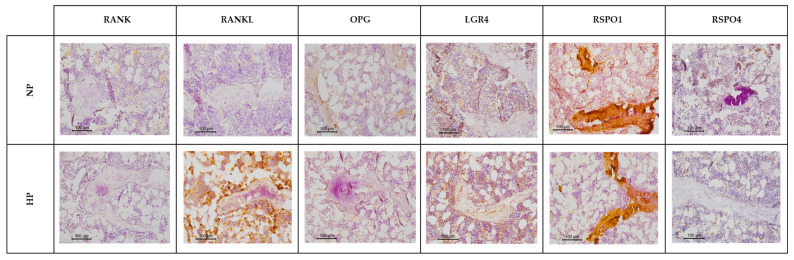
Representative images illustrating the effects of dietary phosphorus intake on the presence of RANK, RANKL, OPG, LGR4, RSPO1, and RSPO4 proteins in tibia. NP: normal phosphorus diet (0.6% phosphorus), and HP: high phosphorus diet (0.9% phosphorus). Each inset indicates the relative scale in µm.

In line with the gene expression data, immunohistochemical analysis of RANK revealed no marked differences between rats fed a NP diet and those on a HP diet. Although a trend toward a decrease in OPG was observed in the HP diet group, it was not statistically significant. However, a significant increase in RANKL and LGR4 levels was detected, along with a decrease in RSPO1 (which was significant when excluding the trabeculae) and RSPO4 levels was observed in the HP diet group (Table 3 and Figure 5).

### 3.6. Influence of High Dietary Phosphorus on Circulating Levels of RANKL, OPG, LGR4, RSPO1, and RSPO4

Serum levels of LGR4 and OPG did not show statistically significant differences between the two dietary groups. However, rats fed the HP diet exhibited a significant increase in serum RANKL levels and a significant decrease in RSPO1 and RSPO4 levels (Table 4).

## 4. Discussion

This study provides compelling evidence that chronic elevation of dietary phosphorus in rats with normal renal function induces significant bone deterioration, particularly affecting trabecular bone microstructure. This deterioration was mechanistically linked to alterations in the RANK/RANKL/OPG signalling pathway and its related components, LGR4, RSPO1 and RSPO4. These findings underscore the potential detrimental impact of excessive dietary phosphorus on skeletal health, even in the absence of renal impairment.

Phosphorus, an essential nutrient, is crucial in several physiological processes, notably as a key component of hydroxyapatite (Ca_10_(PO_4_)_6_(OH)_2_), the primary mineral constituent of bone [21]. Phosphorus is naturally prevalent in many food sources, such as meats, nuts, seeds, legumes, and grains; however, the extensive incorporation of inorganic phosphate additives into processed foods substantially elevates dietary phosphorus consumption. Consequently, clinical phosphorus deficiency is exceedingly rare, and typical dietary intakes often substantially exceed recommended levels [22]. Dietary phosphorus requirements vary depending on age, sex, and individual health conditions. The general recommendation for a healthy adult is 700 mg per day, a level that is easily achieved through the diet, unlike calcium or vitamin D. Notably, the average phosphorus intake among U.S. adults over 20 years of age is reported to be approximately twice the recommended dietary allowance (RDA), highlighting a potential public health concern [23]. Consumption of cola beverages, a significant source of phosphorus, has been consistently associated with adverse effects on bone metabolism, including reduced BMD and increased fracture risk, in both human [24,25,26,27] and animal studies [28,29]. However, while these studies observed an elevation in serum phosphorus, they cannot definitively conclude whether the effects on bone are direct or mediated through its regulatory pathways.

The maintenance of phosphate equilibrium is achieved through a sophisticated, interconnected network of organs, including the kidneys, parathyroid glands, intestines, and skeletal tissue. This system is primarily governed by the hormonal interplay of PTH, FGF23, and 1,25-dihydroxyvitamin D [30]. When serum phosphate levels transiently rise, PTH secretion is triggered, which, in turn, stimulates the renal conversion of 25-hydroxyvitamin D to its active form, 1,25-dihydroxyvitamin D, via the CYP27B1 (1α-hydroxylase) enzyme. Subsequently, both PTH and 1,25-dihydroxyvitamin D induce the production of FGF23 by osteocytes. The combined action of PTH and FGF23 facilitates increased renal phosphate elimination, thereby re-establishing phosphate balance [31].

The results of this study, conducted in rats with normal renal function, align with the established findings regarding phosphate homeostasis. As expected, rats fed a high-phosphorus diet exhibited a significant elevation in serum phosphate, which subsequently triggered a twofold increase in serum PTH levels, likely facilitating the renal excretion of excess phosphate. However, contrary to expectations, a concomitant rise in FGF23 [32] was not observed, despite the increase in serum calcitriol. This observation parallels the early stages of chronic kidney disease (CKD), where PTH typically increases before FGF23 [33], although some controversy exists regarding this temporal sequence [34]. PTH has been described to respond rapidly to declines in renal function and alterations in calcium and phosphate homeostasis [35], a phenomenon supported by the phosphate-induced PTH increase observed in the present study, underscoring its role as a primary regulator. The results suggest that FGF23, while also responsive to elevated phosphate levels, may require a more sustained or distinct stimulus to elicit a significant increase. Alternatively, this may indicate a differential regulatory mechanism between normal renal function and CKD.

While most research on hyperphosphatemia has focused on patients or animal models of CKD, emerging clinical evidence underscores its broader implications for bone health. Notably, a recent observational study in haemodialysis demonstrated that elevated serum phosphate levels were independently associated with an increased risk of bone fractures [36]. Furthermore, the use of phosphate binders was correlated with a reduced incidence of fractures, indicating a potential protective role in maintaining bone integrity [37]. In the CKD animal models, elevated phosphate concentrations are complex with serum calcium, which reduces ionised calcium levels. This reduction, in turn, initiates parathyroid hormone (PTH) release, leading to secondary hyperparathyroidism. This induces a high bone turnover state, mobilizing calcium from bone to re-establish normal serum calcium concentrations. In rats with renal failure, a high phosphorus diet has been shown to induce changes in bone microstructure, affecting both the cortical and trabecular bone compartments, accompanied by increased serum PTH and secondary hyperparathyroidism [38]. Similar bone changes were observed in CKD mice fed a high-phosphorus diet [39]. Previous studies from our group using parathyroidectomised rats with CKD, which were fed a high-phosphorus diet and had a subcutaneous pellet implant releasing normal (non-pathological) PTH concentrations, showed that despite similar serum phosphorus levels, the controlled PTH levels reduced the increase in cortical porosity and preserved the trabecular bone [40]. This demonstrates that in chronic renal insufficiency, both trabecular and cortical bone are affected, and that phosphorus effects are direct and indirect, the latter mediated by PTH. Consistent results were observed in a study involving CKD rats fed diets with low, normal, or high phosphate content (1.2% phosphorus in the high-phosphate diet). In that study, phosphate overload induced hyperphosphatemia and changes in bone microarchitecture, but it notably highlighted the importance of adjusting PTH levels [41]. However, the present study in rats with normal renal function showed that high phosphorus intake, in addition to reducing BMD, particularly affected the trabecular bone, partially resembling the bone loss observed in postmenopausal osteoporosis. Unlike aging, where reduced bone formation and microdamage accumulation lead to progressive trabecular number and size reduction [42,43], postmenopausal osteoporosis, driven by estrogenic deficiency, induces RANKL and decreases OPG, causing increased resorption with selective horizontal trabecular loss and vertical trabecular thickening [44]. This thickening reflects biomechanical adaptation (Wolff’s Law), where remaining trabeculae thicken in response to mechanical loads [45]. Forces redistribute to the remaining vertical trabeculae, inducing hypertrophy to maintain bone strength. However, this compensatory thickening is insufficient to counteract overall bone mass loss, increasing fracture risk.

As previously mentioned, hyperphosphatemia triggers PTH levels, making it challenging to separate their effects. PTH plays a central role in regulating the RANK/RANKL/OPG system, controlling bone remodelling by inducing osteoblast RANKL synthesis and downregulating OPG production, both favouring osteoclastogenesis and bone resorption via a Protein Kinase A (PKA)-driven mechanism [46,47,48]. Indeed, PKA agonists mimic PTH regulation of RANKL and OPG genes [47,49]. However, direct phosphorus effects on this system are less studied. An in vitro study showed that inorganic phosphate dose-dependently reduced RANKL-induced osteoclastic differentiation in human peripheral blood mononuclear cells (PBMC), and mouse macrophage-like cell line RAW 264.7 cells [50]. Another study reported that high phosphorus concentrations increased OPG RNA levels in RAW 264.7 cells, inhibiting osteoclastogenesis in vitro [51]. Conversely, our in vivo studies showed that high phosphorus intake in animals with normal renal function increased RANKL expression and reduced OPG expression. This suggests that PTH effects might predominate over phosphorus effects, but in vitro experiments in UMR-106 osteoblast cells confirmed the phosphorus effect (independent of whether phosphate was combined with PTH).

Focusing on less studied pathway members, LGR4—also known as GPR48—is a RANKL receptor [11] that counteracts RANKL-driven osteoclastogenesis. The extracellular domain of LGR4 directly interacts with RANKL, effectively competing with RANK and consequently suppressing osteoclast differentiation and bone resorption [11]. LGR4 is also present on osteoblasts, where the effect of LGR4 stimulation is that it induces Wnt/β catenin signalling [13]. LGR4 thereby promotes bone formation by enhancing osteoblast maturation and subsequent mineralization. Evidence from murine models demonstrates this, as LGR4 deletion in mice results in delayed osteoblast differentiation and impaired mineralization during embryonic bone formation. Postnatal bone remodelling is also impaired in LGR4 (−/−) mice, exhibiting decreased osteoid production and elevated osteoclast activity, which collectively contribute to a reduction in bone mineral density (BMD) [11]. In humans, LGR4 nonsense mutations are associated with low BMD and osteoporotic fractures [52]. Surprisingly, LGR4 expression increased in our in vivo and in vitro results, possibly reflecting a protective mechanism in bone. This upregulation of LGR4 and RANKL may be related to previous findings showing that in osteoclasts, the RANKL–LGR4 interaction activates G protein-mediated signalling via Gαq/GSK3β, resulting in NFATc1 phosphorylation, which prevents its nuclear translocation and thereby suppresses osteoclastogenesis [12].

LGR4 further interacts with RSPOs, which are known regulators of Wnt signalling [13,14]. These interactions result in the formation of complexes with other Wnt modulators, such as Frizzled/Lrp [53], thereby augmenting the canonical Wnt/β-catenin pathway to facilitate osteoblast differentiation and maturation. Our study showed that phosphorus reduced RSPO1 and RSPO4 levels in vivo and in vitro, while PTH had no significant effect in vitro. However, RSPO2 and RSPO3 did not exhibit significant changes in the in vitro and in vivo studies. These findings suggest a novel mechanism through which phosphorus impairs osteoblast differentiation and maturation, affecting bone homeostasis.

Due to the limitations of traditional bone remodelling biomarkers, including low specificity, sensitivity, and inconsistent findings, the identification of new, more accurate biomarkers for bone alterations is imperative [54]. The determination of the classic marker alkaline phosphatase (ALP) requires only a simple blood drawing and is often a routine part of blood tests. Abnormal levels of ALP in blood usually indicate bone disorders, among others. Also, serum ALP has been correlated with BMD in different studies [55,56]. However, in our study, the rats which were fed a high phosphorus diet showed low BMD, but no significant changes in ALP serum levels (U/L, mean (SD) 77.40 (17.62) vs. 80.00 (19.94); *p* = 0.7609). In contrast, the serum level of RANKL was clearly upregulated. The established role of OPG and RANKL as circulating biomarkers has been widely reported, with the RANKL/OPG ratio particularly emerging as a significant metric for assessing bone remodelling status and bone mass [57]. Clinical observations demonstrate that postmenopausal women with diminished BMD typically exhibit lower serum OPG levels and an increased RANKL/OPG ratio compared to their counterparts with healthy BMD [58]. Furthermore, in patients with rheumatoid arthritis, osteoporosis is associated with decreased serum OPG and elevated RANKL levels when contrasted with patients possessing normal BMD [59]. Our study showed that high phosphorus intake increased serum RANKL without significant changes in OPG. Although a previous study proposed the feasibility of detecting LGR4 in serum [60], here we found no differences in the LGR4 serum levels between the diet groups, despite the bone changes. Interestingly, we found that the serum levels of RSPO1 and RSPO4 were significantly decreased in the rats with high phosphorus intake and trabecular bone deterioration, suggesting their potential as biomarkers for BMD. However, further studies are required to confirm their utility as biomarkers.

Two studies [61,62] have investigated the effect of reducing phosphate additive intake in haemodialysis patients with elevated serum phosphate levels (≥5.5 mg/dL). Both studies found that dietary education, focused on avoiding phosphate additives, resulted in a significant decrease in serum phosphate levels compared to control groups receiving standard care. These results are encouraging and demonstrate how proper nutritional education and follow-up (a cost-effective approach for public health systems) could yield highly beneficial outcomes in controlling serum phosphorus levels and mitigating associated alterations, such as the bone abnormalities observed in the present study.

## 5. Conclusions

This study provides compelling evidence that chronically elevated dietary phosphorus intake in rats with normal renal function induces significant bone deterioration, with a particular impact on trabecular bone microstructure. Mechanistically, this effect is associated with alterations in the RANK/RANKL/OPG signalling pathway and its related components, LGR4, RSPO1, and RSPO4, which warrant further investigation as potential biomarkers for the early detection of bone deterioration. Given these findings and considering the widespread consumption of phosphorus-rich processed foods in industrialised nations, public health initiatives aimed at providing nutritional education on the potential skeletal risks associated with excessive dietary phosphorus are of paramount importance.

## Figures and Tables

**Table 1:** Serum and urinary biochemical parameters of rats fed a normal phosphorus diet (NP, 0.6% phosphorus) and a high phosphorus diet (HP, 0.9% phosphorus).

	NP	HP	*p*
*n*	8	10	
Creatinine clearance (mL/min)	2.92 [2.76, 3.21]	2.72 [2.20, 3.15]	0.248
Serum phosphate (mg/dL)	3.78 [3.45, 4.04]	4.36 [4.08, 4.59]	**0.037**
Serum calcium (mg/dL)	10.19 [10.00, 10.32]	10.11 [9.98, 10.31]	0.823
Serum PTH (pg/mL)	234.43 [179.52, 314.21]	407.70 [374.72, 579.11]	**0.003**
Calcitriol (pg/mL)	5.00 [5.00, 5.41]	15.90 [13.48, 18.18]	**<0.001**
FGF23 (pg/mL)	55.41 [38.57, 89.70]	72.09 [41.91, 78.45]	0.773
FE phosphate %	0.13 [0.07, 0.54]	23.02 [20.22, 26.19]	**<0.001**
FE calcium %	0.69 [0.53, 0.84]	0.27 [0.22, 0.30]	**0.008**

*n*: number of rats. FE: Fractional excretion calculated as [(urine molecule * serum creatinine)/(serum molecule * urine creatinine)] × 100. Data are presented as median [interquartile range]. Bold values indicate statistically significant differences.

**Table 2:** Effect of dietary phosphorus intake on bone parameters measured by micro-CT in tibia.

	NP	HP	*p*
*n*	8	10	
BMD (g/cm^3^)	0.41 [0.35, 0.42]	0.31 [0.31, 0.38]	**0.004**
TMD (g/cm^3^)	1.22 [1.20, 1.23]	1.20 [1.20, 1.22]	0.470
Trabecular structure			
Tb.N (mm^−1^)	3.04 [2.70, 3.17]	2.34 [2.19, 2.83]	**0.006**
BS/TV (mm^−1^)	10.75 [9.69, 11.01]	8.47 [7.72, 10.03]	**0.006**
Conn.Dn (mm^−3^)	171.29 [146.10, 182.75]	100.74 [89.48, 139.88]	**0.014**
BV/TV (%)	22.27 [17.88, 23.00]	18.03 [16.97, 20.82]	**0.030**
Tb.Th (µm)	71.85 [70.33, 73.44]	74.48 [72.91, 77.86]	**0.011**
Tb.Sp (µm)	226.04 [214.61, 249.20]	290.22 [247.72, 329.97]	**0.004**
Tb.Po (%)	77.73 [77.00, 82.12]	81.97 [79.18, 83.03]	**0.030**
SMI	1.22 [1.18, 1.33]	1.36 [1.30, 1.51]	**0.037**
Tb.Pf	9.88 [9.69, 11.68]	11.10 [10.23, 12.26]	0.425
Cortical structure			
Ct.Th (µm)	673.05 [622.70, 690.59]	644.97 [613.89, 655.72]	0.382
Ct.Po.Dm (µm)	113.75 [58.52, 157.87]	94.65 [64.02, 138.48]	0.909
Ct.Po (%)	0.82 [0.29, 1.19]	0.42 [0.33, 0.73]	0.382
BV/TV (%)	99.18 [98.81, 99.71]	99.58 [99.27, 99.67]	0.382
BS/BV (mm^−1^)	5.47 [5.20, 5.68]	5.42 [5.34, 5.65]	0.909

*n*: number of rats. NP: normal phosphorus diet (0.6% phosphorus), and HP: high phosphorus diet (0.9% phosphorus). BMD: Bone Mineral Density; TMD: Tissue Mineral Density; Tb.N: Trabecular Number; BS/TV: Trabecular Bone Surface Density; Conn.Dn: Connectivity Density; BV/TV: Trabecular Bone Volume/Total Volume; Tb.Th: Trabecular Thickness; Tb.Sp: Trabecular Separation; Tb.Po: Trabecular Porosity; SMI: Model Index, Tb.Pf: Trabecular Pattern Factor; Ct.Th: Cortical Thickness; Ct.Po.Dm: Cortical Separation; Ct.Po: Cortical Bone Porosity; BV/TV: Cortical Bone Volume; and BS/BV: Bone surface density. Data are presented as median [interquartile range]. Bold values indicate statistically significant differences.

**Table 3:** Effect of dietary phosphorus intake on RANK, RANKL, OPG, LGR4, RSPO1, and RSPO4 immunostaining in tibia.

	NP	HP	*p*
*n*	8	10	
RANK (%)	0.84 [0.14, 2.29]	0.41 [0.08, 1.34]	0.465
RANKL (%)	4.61 [2.07, 15.94]	34.07 [12.75, 41.62]	**0.021**
OPG (%)	1.48 [0.84, 2.04]	0.84 [0.65, 1.08]	0.150
LGR4 (%)	7.64 [4.48, 8.26]	13.70 [13.20, 17.24]	**0.002**
RSPO1 (%)	22.63 [20.43, 32.06]	19.94 [17.65, 20.92]	0.153
RSPO1* (%)	9.54 [8.99, 15.94]	2.41 [1.72, 2.85]	**0.003**
RSPO4 (%)	5.86 [5.41, 11.11]	3.55 [3.01, 3.94]	**0.011**

*n*: number of rats. NP: normal phosphorus diet (0.6% phosphorus), and HP: high phosphorus diet (0.9% phosphorus). Data represent the percentage of the stained area in immunohistochemistry and are presented as median [interquartile range]. Bold values indicate statistically significant differences. RSPO1* indicates total positive quantification excluding the trabecular surface.

**Table 4:** Effect of dietary phosphorus intake on serum levels of RANKL, OPG, LGR4, RSPO1, and RSPO4.

	NP	HP	*p*
*n*	8	10	
Serum RANKL (pg/mL)	16.00 [11.38, 18.45]	26.74 [23.43, 38.28]	**0.039**
Serum OPG (pg/mL)	2301.50 [2015.20, 3899.15]	3191.60 [2400.80, 4927.60]	0.315
Serum LGR4 (ng/mL)	3.08 [2.35, 3.67]	2.67 [1.91, 2.99]	0.248
Serum RSPO1 (ng/mL)	0.40 [0.34, 0.46]	0.21 [0.16, 0.31]	**0.003**
Serum RSPO4 (pg/mL)	137.90 [120.60, 166.83]	80.15 [71.58, 111.92]	**0.002**
Serum RANKL/OPG (%)	0.61 [0.36, 0.88]	0.60 [0.49, 1.04]	0.479

*n*: number of rats. NP: normal phosphorus diet (0.6% phosphorus), and HP: high phosphorus diet (0.9% phosphorus). Serum protein levels were determined by ELISA. Data are presented as median [interquartile range (IQR)]. Bold values indicate statistically significant differences.

## Data Availability

The data underlying this article will be shared upon reasonable request to the corresponding authors due to privacy reasons.
